# Memoir on the Advantages and Practicability of Dividing the Stricture in Strangulated Hernia, on the outside of the Sac

**Published:** 1833-10

**Authors:** 


*Memoir on the Advantages and Practicability of dividing the Stricture in Strangulated Hernia, on the Outside of the Sac. With Cases and Drawings.* By C. Aston Key, Senior Surgeon to Guy's Hospital. London. 8vo. pp. 161.

The author of this volume has long been known to the profession as one of the best operating surgeons of the day. The subject on which it treats is of great practical importance, and one which it is hardly possible to do justice to in the limited observations of a review. In order to substantiate the general practicability of the operation, he details thirteen fatal cases of hernia, in which the sac was divided after the usual manner. Although, upon a perusal of them, we see no circumstances which would have afforded any valid objection against the external division of the stricture, we feel nevertheless bound to add, that it does not appear to us at all clear that their fatality resulted from laying open the sac; for, of the thirteen cases adduced, it is clear that five died from mortification, consequent, in all probability, upon delay in operating; two from doubtful causes; and six only from peritoneal inflammation. We confess likewise that we should have been better satisfied if the treatment of these last had been given somewhat more in detail; and especially if our author had mentioned the quantity of blood which was taken away; for we believe that peritonitis, from whatsoever cause it may have arisen, is often permitted to terminate fatally from a too sparing use of the lancet.

After giving an "historical sketch" of the operation, which was first performed by Petit, in 1718, Mr. Key adduces the arguments in favour of its performance, as mentioned by Monro, Sir A. Cooper, M. Boyer, and others; and, in connexion with this part of the subject, remarks,

"A prominent character of the operation, and one that raises it above many of the objections that have been brought against it, is, that should the attempt to execute it fail, either from want of dexterity on the part of the operator, or from any peculiar difficulty in the case, the operation can be completed in the ordinary way, by laying the sac open. A surgeon may possibly find great and insuperable difficulty in dividing the stricture externally to the sac; or, having divided the stricture, he may be unable, by the best directed efforts, to return the contents of the hernial tumor: in such a case, he has not brought himself into any dilemma by his unsuccessful attempt; the operation may proceed, as if it had not been made; and neither patient nor surgeon are in a worse position than if the sac had been opened in the first instance, without the attempt to preserve it entire. It is no slight recommendation of the operation, that its failure involves the surgeon in no embarrassment, but leaves him at liberty to adopt the old mode of operation.

"But an intermediate line of proceeding remains for his choice, between the division of the stricture on the outside of the sac, and the laying the latter open in the usual way, so as to expose its contents; namely, the operation, as recommended and practised by Monro, in his fourth case, of making an opening into the sac below the stricture, introducing a director into the opening, conveying it under the stricture, and dividing the latter upon it. Such a proceeding diminishes the danger of inflammation, by less freely exposing the surface of the intestine; and in a simple case of recent enterocele, strangulated, appears to be free from objection. An unusual degree of tightness in the stricture at the inner ring, in inguinal hernia, rendering it impossible to divide the tendon without wounding the neck of the sac, or a thickening of the neck of the sac in femoral hernia, might render this modification of the operation expedient, and perhaps these are the only cases in which it can be considered to be strictly necessary, or indeed to possess any decided advantages. If the stricture could not be relieved without passing the bistoury within the neck of the sac, the operation would be preferable to the more free incision of the sac; but it would not render the return of the contents of the sac more easy than if the stricture were divided on the outside of the peritoneum. It also equally conceals from examination the state of the intestine and omentum, and precludes the possibility of knowing whether they are in a condition to be returned into the abdomen. To these latter objections it is equally liable with the external division of the stricture." (P. 47.)

A few pages farther on he adds:

"I need not again urge the main benefit derived from the external division of the stricture, in the non-exposure of the patient to those causes of inflammation to which the ordinary operation subjects him, and, as experience frequently proves, with most unhappy consequences. The exposure of a bowel in a state of incipient or active inflammation, the handling it in this susceptible state; the incision made into a peritoneal bag already disposed to, if not in an actual state of inflammation, are, as every surgeon will admit, and as his forcible efforts to reduce the hernia without the knife prove that he feels them to be, dangers of no ordinary magnitude to a patient labouring under a strangulated intestine. I do not feel that I have exaggerated the risk of inflammation; for frequently enteritis comes on when, at the time of the operation, the bowel appears to be healthy, and the abdomen free from tenderness; and, when general inflammation precedes the operation, the release of the intestine by the knife rarely succeeds in checking it.

"Cases are sometimes met with in which the patient appears to be doing well after the operation, the evacuations being free and natural, and the sickness and pain subsiding; but, after the lapse of two or three days, the powers begin to sink; the abdomen, though not very tense, is uneasy under pressure, the pulse small and quick, and the tongue becomes dry and coated. This condition is perhaps protracted for several days, and the patient at length dies. A post-mortem inspection discovers the cause of death in the dark colour and lacerable condition of the strangulated portion of bowel, and the vascular state of the surrounding parts.

"This unexpected termination of a case, when it does occur, usually takes place in patients of enfeebled constitution, whose powers are unequal to the restoration of the healthy circulation in the strangulated bowel after its release from the stricture; and in whom, therefore, a slight degree of inflammation gradually ends in the extinction of its vitality. At the period of the operation the intestine, when exposed, presents none of the usual indications of present or approaching gangrene; no infiltration of its tissues, no discoloration beyond that which retarded circulation in a healthy bowel produces, no lack of peritoneal lustre, and no lacerability of texture; it in no point appears to differ from those cases of strangulation in which an early operation is had recourse to before severe symptoms come on, and in which a favourable prognosis is verified by a rapid convalescence. Exposure of a portion of bowel possessing such feeble powers of resistance to morbid influence cannot but tend to increase, probably to excite, a disposition to inflammation; which, though low in degree, is sufficient to destroy its vitality; and it may therefore be fairly regarded as the main agent in the production of gangrene.

"In cases in which great depression of the powers are observed to precede the operation, death sometimes rapidly takes place without any other obvious cause than the exposure of the bowel. The condition of the patient is often found to be manifestly worse after the operation, and stimulants are obliged to be plentifully administered, in order to sustain the sinking powers of life. This may happen without inflammation of the abdominal cavity, or gangrene of the bowel, and is attributable solely to the depressing effect of the operation." (P. 51.)

These copious extracts will clearly show Mr. Key's reasons for giving a preference to this operation over that in ordinary use. In the validity of his views we in some points fully concur, though we think he has laid rather too great a stress on the effect of atmospheric influence on the "exposed bowel," and the danger of dividing the epigastric artery; but we must refer our readers to the work itself for a more extended examination of this part of the subject.

After giving the arguments which have been brought forward in favour of and against the operation, he details his "own experience of it, and the steps of the operation in the three most common forms of the disease." With a candour which cannot be too much praised, he gives the particulars of the first two instances in which he attempted it, and in both of which he was ultimately compelled to lay open the sac. Great merit is due to him for the plain and strait-forward manner in which these cases are mentioned, and which, we ought in fairness to add, offer no valid objection to the general adoption of the operation. When operating upon femoral hernia,

"The extent, as well as the form of the incision through the integuments, may seem of minor importance, except as far as it tends to facilitate the after steps of the operation; yet it may be as well to disturb the subjacent cellular membrane as little as possible, as inflammation is less likely to follow, and to assume the form of erysipelas. For this reason, the inverted T incision, usual in the operation for femoral hernia, may be in most cases reduced to a single incision, either at right angles to Poupart's ligament, or in a transverse direction across the tumor. In patients who are spare, and in whom the neck of the sac lies at no great depth from the surface, it is unnecessary to disturb the cellular membrane by turning aside the flaps of the integument. This will diminish the suppurative inflammation, and in such cases will afford ample room for the operation. I have not made trial of the perpendicular form of incision, but a single transverse one I have found sufficient, when the integuments have been loose, and the tumor not large. The superficial fascia adheres firmly to the common integuments, and is usually turned aside with them, especially when the latter are pinched up for the purpose of making the first incision. The fascia propria is therefore quickly exposed, and forms the first distinct covering of the tumour, being darker than the more superficial cellular investment. It is under the outer layer of this fascia that the adipose structure is formed, and which often assumes the appearance of omentum. The director easily makes its way under this fatty matter as far as the neck of the sac, which lies deeper than the operator at first supposes. The point of the director should be applied rather to the inner than to the outer part of the neck of the sac, as it will be found more easily to pass under the stricture at this part. It should not at first be attempted to be thrust under the stricture, as the firmness of the parts forming the stricture would resist it. But the seat of stricture being felt, the operator should depress the end of the director upon the sac, which will yield before it, and then, by an onward movement, the director slides under the stricture." (P. 142.)

"In inguinal hernia the incision may be so conducted as to enable the surgeon to divide the stricture either at the internal or at the external ring. The opening in the skin must be made higher than is usual in the ordinary operation on a bubonocele. The incision should begin at the neck of the tumor, or where it seems to quit the abdomen, and should be continued downward for about an inch and a half. This will lay bare the lower portion of the external oblique tendon, where it forms the ring. A small opening should then be made in the tendon, just above the ring, sufficient to admit the end of the director, which will enable the operator to ascertain if the stricture be at the lower or upper opening. The size of the hernia, and the length of time it has existed, will, in some measure, serve to guide him; but he may immediately decide the point by passing the director downward under the edge of the external ring, and feeling whether it embraces the tumour firmly or not; or, by making pressure on the swelling below, he may feel if the fluid contents of the tumor can be forced upward above the ring, so as to distend the sac in the inguinal canal. This point being decided, if the stricture be at the lower ring, he has only to pass his director under its margin, and to divide it to a sufficient extent.

"If the stricture exists higher up at the neck of the sac, where it will be found in the majority of herniæ of this description, the opening in the tendon should be enlarged to the extent shown in the second drawing, for the purpose of passing the director under the deeper stricture. The lower margin of the two muscles will be brought into view, with some of the descending fibres of the cremaster. These may be separated by disturbing the cellular membrane with the end of the director; and the instrument may then be introduced under the transversalis muscle till it reaches the stricture. In the subject the director, when introduced in this manner, passes before the transversalis fascia: this will diminish what little risk there may be of wounding the peritoneum, and will carry the knife farther from the epigastric artery: the tenuity, however, of this fascia will, perhaps, often allow the director to pass beneath it. The instrument should be depressed upon the sac, in order to carry its point under the border of the transversalis, which may be divided to the extent required. This operation is more difficult than the division of the stricture in femoral hernia: the principal difficulty lies in the accurate separation of the lower edge of the internal oblique muscle, for the easy passage of the director. The stricture, however, is not so firm in inguinal as in femoral hernia, and the introduction of the director under the transversalis tendon will not be difficult, where it is fairly passed up to the neck of the sac before the attempt is made. The steps of the operation will be much the same in those smaller hernia which are lodged in the inguinal canal. When the stricture is divided, a greater degree of pressure will be required to return the contents of a large inguinal hernia, on account of the distance from the neck of the sac to the bottom of the tumour, and especially when the omentum forms a part of its contents.

"In small bubonocele, where the protrusion has scarcely reached the external ring, and accompanied, as it commonly is, with an imperfect descent of the testicle, the same manner of operating may be followed.

"I prefer making the opening in the tendon in the manner described in the drawing to slitting up the external ring, as the opening in the tendon will afterwards unite more firmly if the ring be left entire, and will not dispose the patient more to the disease than when the operation is performed in the usual way."

In umbilical hernia, which Mr. Key states is, of all forms of the disease, the one which most requires the sac to be preserved entire,—

"The division of the tendinous margin of the umbilical aperture is not difficult; it requires care, on account of the extreme thinness of the sac; and the operation, therefore, consists in a cautious exposure of the linea alba, where the tumor emerges from the abdomen. The orifice of the sac is rendered readily accessible at its upper part by the descent of the swelling towards the pubes; the sac, when it emerges from the abdomen, does not extend equally in all directions, but gradually makes its way downwards, in consequence of the weight of its contents; and therefore, in old large herniæ, though the aperture in the tendon bears but a small comparison to the size of the tumor, it is scarcely at all overlapped by it at the upper part." (P. 150.)

In conclusion, we must express our regret that Mr. Key has not furnished us with a greater number of cases, illustrating the advantages of the operation which he advocates. In this most important point our author is extremely deficient; and we cannot help thinking that he would have acted more wisely, if he had delayed the publication of his memoir till experience had enabled him to place it before the profession in a more satisfactory manner.

